# Foreign Immigration

**Published:** 1856-08

**Authors:** 


### FOREIGN IMMIGRATION.

During the year 1854 four hundred and twenty thousand foreigners came to the United States; during last year, 1855, only two hundred thousand—of whom, perhaps, two-thirds landed in New York.

It is this foreign element which makes our city appear so unhealthy as it does. Thousands are landed every year at Castle Garden in a dying condition.

